# Correlation between FAK and EGF-Induced EMT in Colorectal Cancer Cells

**DOI:** 10.1155/2020/5428920

**Published:** 2020-02-17

**Authors:** Kun Huang, Ningning Gao, Donglin Bian, Qixi Zhai, Puxu Yang, Mingwei Li, Xuemei Wang

**Affiliations:** Department of Ultrasonic Diagnosis, The First Affiliated Hospital of China Medical University, Shenyang, Liaoning Province, China

## Abstract

Epithelial-mesenchymal transition (EMT) plays an important role in the invasion and metastasis of colorectal cancer, which is mediated by FAK and EGF. However, whether FAK participates in EMT in colorectal cancer cells through the EGF/EGFR signaling pathway remains unknown. The aim of this study was to investigate the effector mechanisms of FAK in the process of EGF-induced EMT in colorectal cancer cells and to determine whether miR-217 is involved in this process. Caco-2 cancer cells were routinely cultured with and without treatment with 100 ng/mL EGF, and changes in cell morphology were observed using an inverted microscope. In addition, a transwell assay was used to detect cell migration under the condition of EGF treatment. The expression of FAK, pFAK, E-cadherin, vimentin, and *β* actin was assessed by western blotting, and the expression of miR-217 was assessed using real-time PCR. We found that EGF induced EMT in colorectal cancer cells and enhanced cell migration and invasion ability. Moreover, FAK was involved in the EGF-induced EMT of colorectal cancer cells. EGF upregulated the expression of E-cadherin in colorectal cancer cells by activating FAK, and miR-217 was found to participate in EGF-induced EMT in colorectal cancer cells. Our findings indicate that EGF induces EMT in colorectal cancer cells by activating FAK, and miR-217 is involved in the EGF/FAK/E-cadherin signaling pathway.

## 1. Introduction

Colorectal cancer is a common malignancy of the digestive tract, and the metastasis is the main cause of death in colorectal cancer. However, tumor metastasis is a highly complex process involving multiple factors and steps. Recent studies have shown that epithelial-mesenchymal transition (EMT) plays an important role in tumor invasion and metastasis [1, 2]. EMT refers to the phenomenon by which epithelial cells lose their epithelial characteristics and transform into mesenchymal cells under certain physiological or pathological conditions. The changes cause the cells to undergo morphological changes and exhibit increased migration ability. The process of EMT includes not only changes in cell phenotype but also changes in cell markers, such as the loss of epithelial cell markers (e.g., E-cadherin) and gain of mesenchymal cell markers (e.g., vimentin and alpha-smooth muscle actin (*α*-SMA)). Studies have shown that the occurrence of EMT is a complex process involving the tumor microenvironment, growth factors, kinases, transcription factors, and micro-RNAs (miRNAs), but the specific mechanisms underlying this process remain unclear. Many studies are currently carried out to investigate the detailed mechanisms underlying the occurrence of EMT in tumor cells.

Numerous factors can induce EMT in tumor cells including EGF, which initiates EMT in colorectal cancer cells through the EGF/EGFR signaling pathway, thereby facilitating invasion and metastasis. This triggers the phosphorylation of tyrosine kinases, followed by the initiation of a signaling pathway that activates downstream signaling molecules to facilitate EMT. EGF further activates phosphorylase kinases by binding to EGFR, which causes abnormal protein expression and the occurrence of EMT. However, the specific mechanism remains unclear.

FAK is a central hub for various signaling pathways. The activation of FAK regulates a series of signaling pathways and induces EMT in various tumor cells [3], thereby affecting a series of cellular biological behaviors such as proliferation, survival, adhesion, migration, invasion, and metastasis. However, the specific mechanism by which FAK regulates the occurrence of EMT is unclear. Thus far, it is clear that upstream molecules mainly bind to its N-terminus, which causes the rapid phosphorylation of Tyr397 and its subsequent binding to various downstream molecules. This activates the downstream pathway, inducing EMT in colorectal cancer cells [4] and enhancing their migration abilities. However, it is still not clear which proteins and which miRNAs are involved in this process.

MiRNAs are endogenous, noncoding, single-stranded, small-molecule RNAs found in eukaryotes that regulate gene expression. A variety of abnormally expressed miRNA have been detected in tumor tissues and are thought to be involved in the regulation of processes such as tumor cell proliferation, apoptosis, migration, and invasion. These are expected to become new markers for prognosis. It has been reported that miR-217 is highly expressed in breast cancer tissues [5], and there are also reports that its expression is downregulated in colorectal cancer tissues [6]. However, there have been no reports thus far on whether miR-217 is involved in EGF-induced EMT in colorectal cancer cells.

It has not yet been determined whether FAK is involved in the regulation of EMT in colorectal cancer cells through the EGF/EGFR signaling pathway. Therefore, this study aims to investigate whether FAK is involved in the regulation of colorectal cancer cells through the promotion of EMT via the EGF/EGFR signaling pathway and the enhancement of migration ability. We further sought to determine whether miR-217 is involved in this process to provide a theoretical basis for the clinical identification of new targets.

## 2. Materials and Methods

### 2.1. Cell Culture

The human colorectal cancer cell line Caco-2 was cultured in an incubator in RPMI1640 medium containing 100 *μ*g/ml streptomycin, 100 U/ml penicillin, and 10% fetal bovine serum. The incubator was maintained at saturated humidity with 5% CO_2_ and a temperature of 37°C. Cells were routinely passaged once every 3 to 5 days, and 0.25% trypsin was used for cell digestion each time. All experiments were performed on cells in the exponential growth phase.

### 2.2. Transfection of miR-207 Mimics

The *miR-207 mimics* and negative control (GenePharma Corporation, Suzhou, Jiangsu, China) were transfected with lipofectamineTM 3000 transfection reagent. Then, the cells were further cultured for 12 hours and seeded into new plates at an appropriate density per well for the next assay.

### 2.3. Western Blotting

Cells (1 × 10^6^) for the control and treatment groups were added to 6-well plates, and 200 *µ*l cell lysis buffer (purchased from Beyotime) was added. After ultrasonic lysis, the solution was centrifuged at 13,000 rpm for 20 min, and the supernatant was obtained for protein quantification using the BCA method. Next, 3x sample buffer was added, the solution was boiled for 5 min, and the sample was stored in a freezer at −20°C. Resolving gels and stacking gels were prepared, and 20 *µ*l of sample was obtained, containing 50 *μ*g of protein. SDS-polypropylene gel electrophoresis was performed with a stacking gel at 60 V for 60 min and a resolving gel at 120 V for 60 min. The protein was then transferred to a PVDF membrane, which was blocked with 5% skim milk for 1 hour. FAK, pFAK (Try 397), pFAK (Try 925), E-cadherin, vimentin, and *β*-actin primary antibodies were then added, and the membrane was incubated overnight at 4°C. On the next day, the membrane was washed with TTBS three times, and goat anti-rabbit secondary antibody labeled with horseradish peroxidase (1 : 1000) was added, followed by blocking of the membrane at room temperature for 1 hour. The membrane was washed with TTBS for 10 min three times, and ECL luminescence reagent was added. Photos were taken and analyzed using a gel documentation system.

### 2.4. Transwell Assay of Migration and Invasion

For transwell assay with or without matrigel, 2% fetal bovine serum medium was added to the lower chamber of the transwell, after which 2.5 × 10^5^ cells were obtained and added to 1 ml of serum-free cell suspension. After mixing, 200 *μ*l of cell suspension was obtained and seeded in the upper chamber of a transwell with a pore size of 8 *μ*m with or without matrigel. The transwell was incubated in a 37°C incubator for 24 hours. The inner chamber of the transwell was then removed. The remaining cells inside the chamber were removed with a cotton swab, and the chamber was dried at room temperature for 1 hour. The chamber was stained with crystal violet for 15 min, rinsed with tap water, dried, and photographed under a microscope. Five 20x visual fields were chosen in each chamber, and the cells were counted. The number of cells that had crossed the filter membrane indicated the migrating or invasive ability of tumor cells.

### 2.5. Isolation of Total RNA

Isolation of total RNA from cells was performed according to the instructions for the TRIzol reagent. For adherent cells, the culture medium was discarded, PBS was added and cells were washed twice, 1 ml TRIzol was added, and the cells were disassociated by careful and repeated pipetting. The mixture was allowed to stand at room temperature for 5 min so that the cells were lysed thoroughly. Next, 0.2 ml chloroform was added for each ml of TRIzol, and the mixture was vigorously shaken for 15 s and allowed to stand at room temperature for 3 min before centrifuging at 4°C and 12,000 ×*g* for 15 min. The supernatant was obtained and loaded into a new centrifuge tube, and an equal volume of isopropyl alcohol to chloroform was added and mixed thoroughly; this was allowed to stand at room temperature for 10 min and centrifuged at 4°C and 12,000 ×*g* for 10 min, after which the supernatant was discarded. Then, 1 ml of 75% ethanol was added, and the precipitate was gently washed. The mixture was centrifuged at 4°C and 7,500 ×*g* for 5 min, and the supernatant was discarded. The precipitate was air-dried on a superclean bench and dissolved in 20–30 *μ*l of nuclease-free water. For determination of RNA concentration and purity, a small amount of RNA was obtained and diluted, and the RNA concentration of the sample was calculated based on OD260 (a value of 1 at OD260 indicated an RNA concentration of 40 *μ*g/mL). An OD260/OD280 ratio between 1.8 and 2.1 indicated that the sample was of sufficient quality. The sample was stored in a freezer at −80°C.

### 2.6. Reverse Transcription

A reaction mixture of 20 *μ*l containing 4 *μ*l 5x reverse transcription buffer, 1 *μ*l reverse transcriptase, 1 *μ*l oligo dT primer (50 *μ*M), 1 *μ*l random primer (100 *μ*M), 1 *μ*l total RNA (1 *μ*g/*μ*l), and 12 *μ*l RNase-free water was added to an RNase-free EP tube for the reverse transcription of total RNA and synthesis of cDNA. This solution was mixed thoroughly and centrifuged for 10 s. The conditions for reverse transcription were as follows: 16°C for 30 min, 42°C for 30 min, and reverse transcriptase inactivation at 85°C for 5 min. The reaction was stored at −20°C after cooling on ice before use.

### 2.7. Fluorescent Quantitative PCR (RT-PCR)

MiR-217 primers were purchased from Shanghai GenePharma Co., Ltd: upstream primer, 5′ XXXXXXXXXXX 3′ and downstream primer, 5′ XXXXXXXXXXX 3′. The 20 *μ*l reaction mixture contained 10 *μ*l 2x SYBR Premix Ex Taq II, 0.4 *μ*l upstream primer (10 *μ*M), 0.4 *μ*l downstream primer (10 *μ*M), 0.4 *μ*l ROX II, 1 *μ*l cDNA, and 7.8 *μ*l ddH_2_O. The PCR reaction conditions were as follows: denaturation at 95°C for 5 mins; , 40 cycles at 95°C for 30 s, 56°C for 45 s, and 72 for 40 s, followed by 72°C for 10 min.

After the reaction was complete, amplification and dissolution curves were confirmed, and the result was analyzed by the ΔΔCT method. For each PCR reaction, plates with deionized water were used as a negative control, and the experiment was performed in triplicate for each sample.

### 2.8. Statistical Analysis

All data were obtained from experiments performed at least three times, and the results were expressed as the mean ± standard deviation. Statistical analysis of the experimental data was performed using the SPSS 17.0 statistical software. The mean values of the two groups were compared using *t*-tests: ANOVA (parametric) and Tukey's test were used in the comparison of multiple groups. *P* < 0.05 indicated a significant difference.

## 3. Results

### 3.1. EGF Induces EMT and Enhances Migration and Invasion Abilities of Caco-2 Cancer Cells

**Figure 1:**
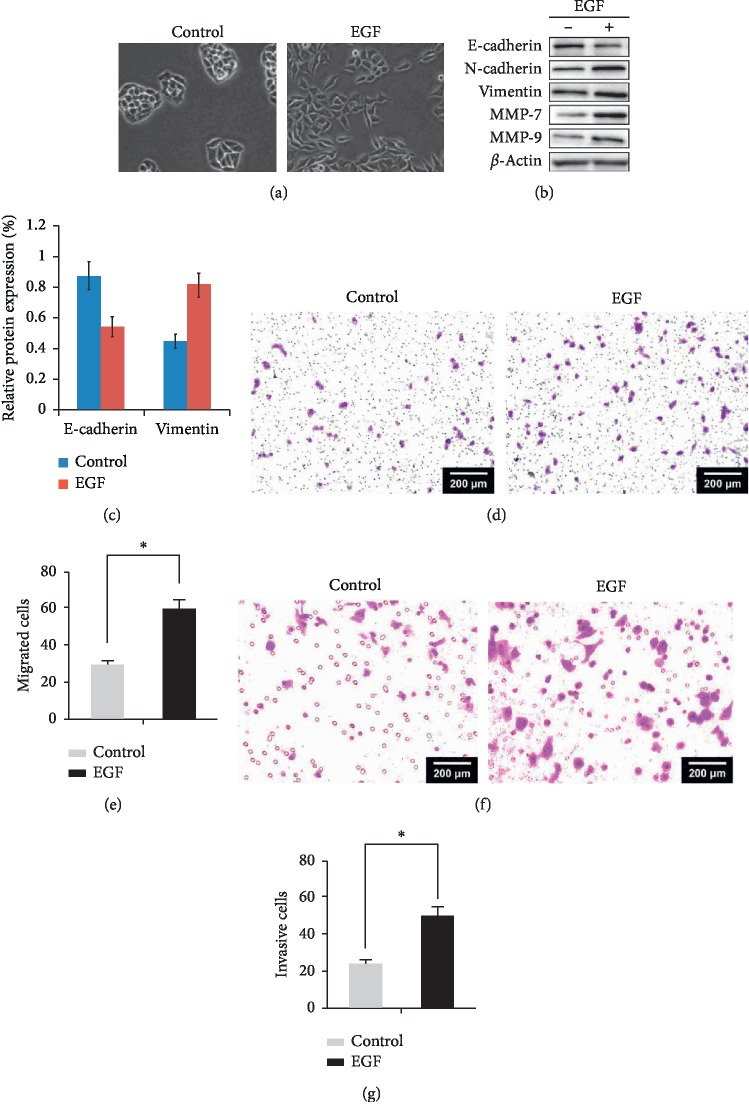
EGF-induced EMT in colorectal cancer cells. Caco-2 cells were treated with 100 ng/mL EGF for 24 hours. (a) Changes in cell morphology were observed using an inverted microscope. (b) Changes in EMT marker proteins were detected by Western blotting. (c) Column graph was used to shown the repeat results of (b). (d, e) EGF was used to treat colorectal cancer cells for 24 hours, and the number of cell migration was detected by transwell assay. (f, g) EGF was used to treat colorectal cancer cells for 24 hours, and the number of cell invasion was detected by transwell assay ^*∗*^*P* < 0.05.

Caco-2 colorectal cancer cells were treated with 100 ng/mL EGF for 24 hours, and changes in cell morphology were observed using an inverted microscope (Figure 1(a)). Following EGF treatment, the cells changed from tightly connected ovals to loosely connected and long fusiforms. Western blotting was used to detect epithelial and mesenchymal proteins (Figures 1(b) and 1(c)). Compared with the control, the expression of E-cadherin was significantly reduced after 24 hours of EGF treatment, while the expression of vimentin was significantly elevated.

Next, cell migration and invasion abilities were assessed in Caco-2 cells treated with 100 ng/mL EGF for 24 hours with a transwell assay. The results showed that compared with the control cells, Caco-2 cancer cells treated with EGF exhibited a higher number of cells migration and invasion per visual field than the control (30 ± 2 vs. 60 ± 5, *P* < 0.05, Figures 1(d)–1(g)). This indicated that the migration and invasion abilities of colorectal cancer cells were significantly enhanced by EGF treatment.

### 3.2. FAK Is Involved in EGF-Induced EMT in Colorectal Cancer Cells and Enhances Cell Migration and Invasion Abilities

**Figure 2:**
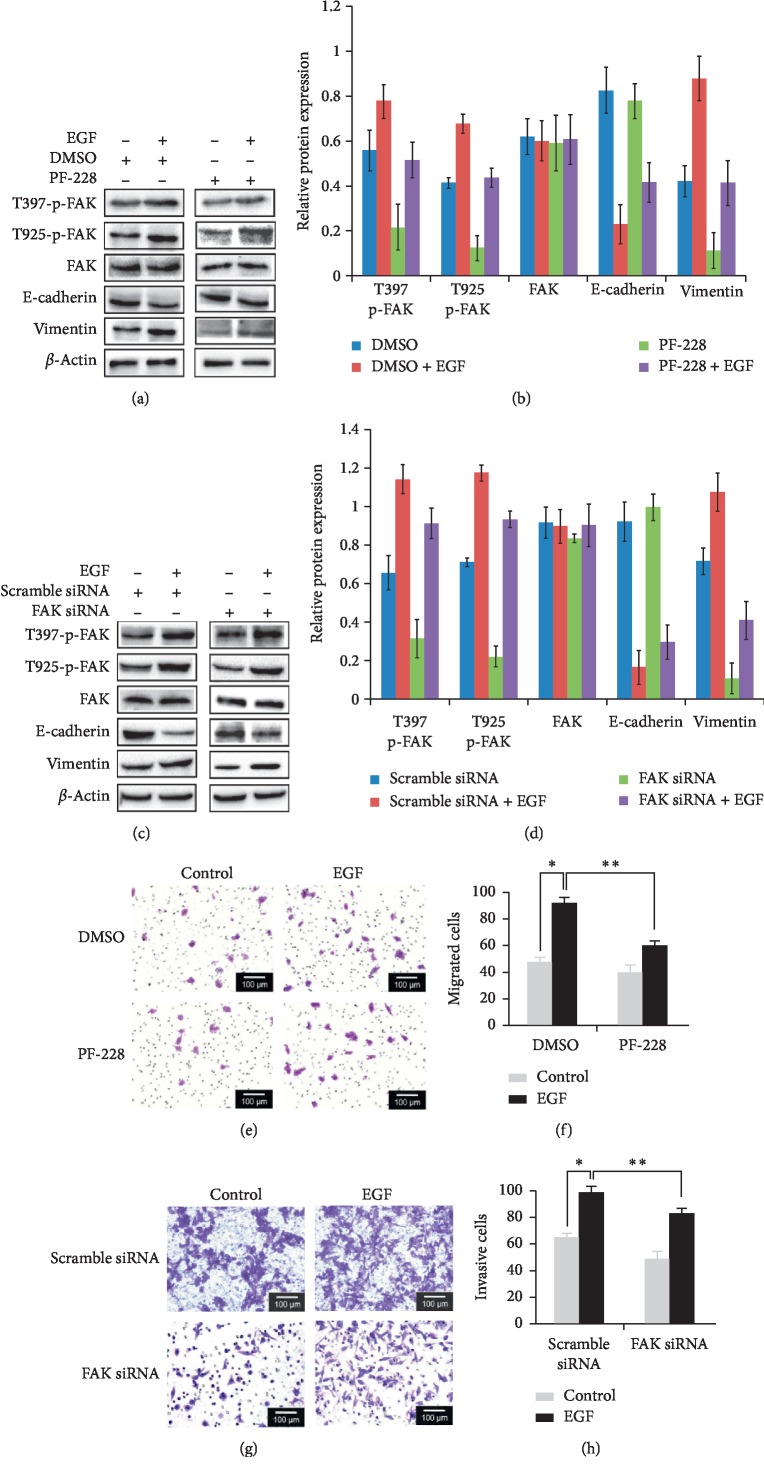
FAK is involved in EGF-induced EMT in colorectal cancer cells and enhances cell migration and invasion abilities. (a, b) Cells were pretreated with FAK inhibitor PF-228 (10 *μ*M) for 1 hour, and the phosphorylation levels of FAK and expression levels of E-cadherin and vimentin were significantly inhibited by the detection of Western blotting. (c, d) Cells were pretreated with FAK siRNA, and the phosphorylation levels of FAK and expression levels of E-cadherin and vimentin were significantly inhibited by the detection of Western blotting. (e, f) With the treatment of EGF and FAK inhibitor/FAK siRNA, the number of cell migration was detected by transwell assay. (g, h) With the treatment of EGF and FAK inhibitor/FAK siRNA, the number of cell invasion was detected by transwell assay.

After treatment of Caco-2 cells with EGF for 24 hours, FAK phosphorylation was increased, as detected by western blotting. In addition, cells were pretreated with the FAK inhibitor PF-228 (10 *μ*M) for 1 hour and then treated with EGF for 24 hours to observe changes in epithelial and mesenchymal protein expression. Furthermore, cells were also transfected with FAK siRNA and then treated with EGF for 24 hours to observe the protein expression. As a result, EGF-induced downregulation of E-cadherin and upregulation of vimentin were significantly inhibited by pretreatment with FAK inhibitor PF-228 and FAK siRNA, compared with those with EGF treatment alone (Figure 2). The above results suggested that EGF induced the phosphorylation of FAK (397 sites) in colorectal cancer cells, and the the downregulated expression or activity of FAK significantly inhibited EGF-induced EMT in colorectal cancer cells.

After pretreatment with FAK inhibitor PF-228 (10 *μ*M) for 1 hour, the migration and invasion abilities of EGF-induced colorectal cancer cells was determined by the transwell assay. Furthermore, cells were also transfected with FAK siRNA and examined the migration and invasion abilities of EGF-induced colorectal cancer cells. The results showed that the treatment with 100 ng/mL EGF for 24 hours increased the migration and invasion abilities of Caco-2 cells, evidenced by the mean number of invasive cells increasing (*P* < 0.05). Compared with EGF alone, PF-228 or FAK siRNA combined with EGF significantly reduced the mean number of cell migration and invasion (*P* < 0.05). The downregulated expression or activity of FAK inhibited the promoting effects of EGF on the migrative and invasive effect (*P* < 0.05, Figure 2). The above results show that FAK activation is involved in EGF-induced migration and invasion of colorectal cancer cells.

### 3.3. miR-217 Is Involved in EGF-Activating FAK to Induce EMT by Targeting E-Cadherin in Caco-2 Cancer Cells

#### 3.3.1. EGF Induces Upregulation of miR-217 in Caco-2 Cells

**Figure 3:**
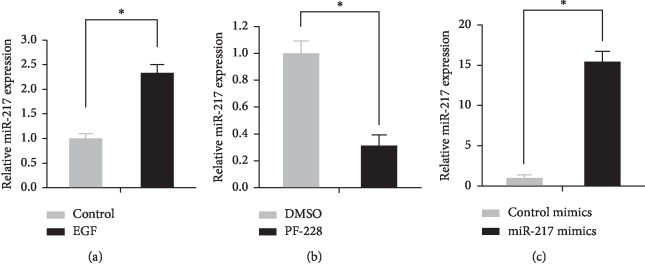
EGF upregulated the expression of miR-217, and FAK inhibitors downregulated miR-217 expression. (a) EGF was used to treat Caco-2 cells for 24 hours, and the expression level of miR-217 was detected by real-time PCR; ^*∗*^*P* < 0.05. (b) With the treatment of PF-228, the expression level of miR-217 was detected by real-time PCR; ^*∗*^*P* < 0.05. (c) With the treatment of miR-217 mimics, the expression level of miR-217 was detected by real-time PCR; ^*∗*^*P* < 0.05.

Caco-2 cancer cells were treated with 100 ng/mL EGF for 24 hours, and the changes in the expression level of miR-217 were assessed using real-time PCR (Figure 3(a)). The results showed that the expression of miR-217 increased after EGF treatment.

#### 3.3.2. FAK Inhibitors Downregulate miR-217 Expression

Caco-2 cancer cells were treated with FAK inhibitor PF-228 (10 *μ*M) for 24 hours, and the expression of miR-217 was detected using real-time PCR (Figure 3(b)). The results showed that after treatment with PF-228, the expression of miR-217 in Caco-2 cells was 0.7 times lower than that in the control group (*P* < 0.05).

#### 3.3.3. Exogenous Overexpression of miR-217 Leads to Downregulation of E-Cadherin by Binding 3′ UTR of E-Cadherin

**Figure 4:**
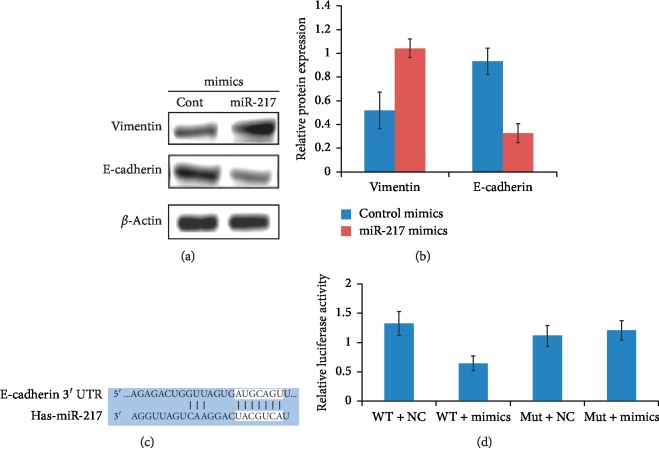
FAK inhibitor PF-228 inhibited EGF-induced upregulation of Snail in colorectal cancer cells. (a, b) Overexpression of miR-217 and detection of changes in E-cadherin expression levels. Lipo2000 was used to transfect Caco-2 cells with miR-217 mimics, and western blotting was used to detect decrease in the level of E-cadherin protein. (c) miR-217 binding site in the 3′ UTR of E-cadherin. (d) Luciferase reporter assay for cotransfection of miR-217 mimics and 3′-UTR of E-cadherin.

Caco-2 cells were transfected with miR-217 mimics and control. After 96 hours, changes in the levels of E-cadherin protein were detected by western blotting (Figures 4(a) and 4(b)). The results showed that the expression of E-cadherin protein in the miR-217 mimics group was downregulated compared with that in the control group. The potential targets of miR-217 was explored using miRWalk 2.0 (http://diana.imis.athena-innovation.gr/DianaTools/index.php?r=microT_CDS/index) and found that E-cadherin was a putative target of miR-217 (Figure 4(c)). Then, the dual luciferase reporter assay was performed to examine miR-217 directly bound with the 3′ UTR region of E-cadherin mRNA. According to the predicted binging site, we structured luciferase reporter plasmids. The pmirGLO luciferase reporter plasmid, containing a wild-type or mutant-type E-cadherin 3′ UTR, was cotransfected with miR-217 agomir or negative control. It showed that luciferase activity of the cells transfected with wild-type plasmid and agomir was downregulated, whereas it had no effect when the cells were transfected with the mutant type (Figure 4(d)).

## 4. Discussion

It has been confirmed that EMT is involved in tumor invasion and metastasis and plays an important role in these processes. In the EMT process, epithelial cancer cells lose their apical-basal polarity and cell-cell and cell-ECM adhesion, accompanied with cytoskeleton reorganization, thus facilitating cancer cell invasion and metastasis [7]. In addition, EMT also endows tumor cells with cancer stem cell-like features and resistance to senescence and apoptosis [8]. For decades, studies have elucidated the essential role of the microenvironment on phenotype conversion exhibited by cancer cells. Especially in CRC, Wnt/*β*-catenin signaling is simultaneously involved in regulating EMT and tumor angiogenesis and is crucial in tumorigenesis and progression. In addition, EMT plays a role in maintaining oxaliplatin resistance [9]. EMT is a complex process involving multiple stages and factors. Studies have shown that EGF can reduce the expression of E-cadherin and enhance the occurrence of EMT in uterine cancer cells [3]. Similarly, EGFR is also highly expressed in colorectal cancer and is associated with the invasion and metastasis of colorectal cancer. EGF further activates phosphorylase kinases by binding to EGFR, which causes abnormal protein expression and the occurrence of EMT. However, it is still not clear which signaling pathways are activated to promote metastasis in colorectal cancers.

FAK is a nonreceptor tyrosine kinase. As a central hub in signal transduction, it is involved in a variety of signaling pathways, activating transcription factors, regulating expression of related genes, and inducing EMT in tumor cells. Studies have shown that FAK enhances the invasive ability of head and neck tumors by inducing EMT in head and neck squamous cells [10]. FAK is also overexpressed in infiltrating metastatic tumors such as colon cancer and liver cancer. The role of FAK lies in the binding of its N-terminus with upstream molecules, which causes rapid Tyr397 phosphorylation and activation of FAK. Phosphorylation can trigger signal transduction mechanisms, activate downstream signaling molecules, and cause corresponding biological responses. EGFR belongs to the tyrosine kinase receptor and can activate FAK by binding to the N-terminus. EGFR activation promotes epithelial mesenchymal migration of human retinal pigment epithelial cells through regulation of the FAK-mediated Syk/Src pathway [11]. Activating the EGFR/FAK pathway promotes migration of A549 lung cancer cells [12]. EGF-induced activation of FAK at Y925 regulates focal adhesion disassembly during cell spreading [13]. This study demonstrated that EGF/EGFR can phosphorylate FAK and induce EMT in colorectal cancer cells.

As knowledge of miRNAs continues to grow, more than 1,000 miRNAs have been identified in human gene banks. In more than 20 types of human malignant tumors, specific differential expression of miRNAs was found [14]. There have been few reports on the relationship between miRNAs and colorectal cancer [15], but research in this area is ongoing. miR-30 is downregulated by oncogenic signals such as epidermal growth factor (EGF). Epidermal growth factor (EGF) has been shown to be one of the most potent inducers of EMT in cervical cancer and associated with cervical stromal invasion and nodal metastasis. Chronic EGF treatment induces EMT via upregulation of EMT-inducing transcription factor Snail in cervical cancer cells, and EGF-mediated EMT is correlated with EGF receptor (EGFR) overexpression and clinical progression of cervical cancer. A study by Balaguer et al. showed that miR-137 expression was low in colon cancer and its promoter was hypermethylated [16]. Ng et al. [17] observed high expression of miR-21 and low expression of miR-143 in colon cancer. These two miRNAs are negatively correlated and both are closely related to liver metastasis of colon cancer. Studies by Wu et al. [18] showed that miR-107 could negatively regulate the growth of colorectal cancer, angiogenesis, and other processes by regulating HIF-1*α* expression. miRNAs can downregulate the expression of E-cadherin by specifically binding to IGFlR, FAK, and other proteins. This enhances EMT in cells, thereby affecting cell adhesion and motility. The process of tumor cell metastasis also involves this behavior. However, there have been few reports on miR-217 thus far, and only a small number of researchers have conducted relevant studies. For example, miR-217 is expressed in breast cancer, leukemia [19], lung cancer [20], gastric cancer [21, 22], colorectal cancer [23–25], and pancreatic cancer [24–29]. It exhibits low expression in gastric cancer and is significantly downregulated in colorectal cancer [6]. In addition, miR-217 can serve as a therapeutic target for breast cancer [5] and can also inhibit the growth, migration, and invasion of triple negative breast cancer cells [30]. In this study, the role of miR-217 in colorectal cancer cells was further explored, and we found that EGF upregulates miR-217 by activating FAK, causing EMT in colorectal cancer cells and thereby enhancing the invasion and metastasis of colorectal cancer.

The results of this study showed that EGF induced EMT in Caco-2 cancer cells and enhanced cell migrating ability. The specific mechanism is the binding of EGF to EGFR, which activates downstream signaling pathways, leading to the downregulation of E-cadherin and upregulation of vimentin, thereby inducing EMT in colorectal cancer cells. E-cadherin and vimentin are very important factors in EMT process of cancer development. Furthermore, they have important relationship with EGF/FAK/miR-207 pathway. So, they were used as the EMT factors to represent the status of EMT. Similar to another study [31], this study further demonstrated that EGF can induce EMT in colorectal cancer cells. Park GB and Kim D reported that VEGF and TGF-*β*1 can increase the expression of EMT markers, and knockdown of FAK blocked VEGF and TGF-*β*1-mediated EMT processes in CSE-stimulated RPE cells [11]. The same phenomenon was found in our research. At the same time, the observed increase in cell migration ability indicates that EGF enhances the metastasis of colorectal cancer cells. FAK inhibitors can also inhibit the downregulation of E-cadherin and upregulation of vimentin in the process of EGF-induced EMT in colorectal cancer cells. This also led to a significant decrease in the number of migrating cells, indicating that FAK is involved in the process of EGF-induced EMT and is related to colorectal cancer invasion and metastasis. As the inhibition of FAK reduces the migration ability of colorectal cancer cells, it provides an approach for subsequent targeted therapy. Moreover, the results of this study showed that EGF induced FAK phosphorylation in Caco-2 cancer cells, and FAK inhibitors could inhibit EGF-induced FAK phosphorylation in colorectal cancer.

Finally, this study showed that miR-217 was involved in EGF-induced EMT. We observed the activation of FAK by EGF and upregulation of miR-217 expression in Caco-2 cancer cells, resulting in the downregulation of E-cadherin and induction of EMT in colorectal cancer cells. miR-217 downregulates E-cadherin expression by binding to 3′ UTR of E-cadherin. The downregulation of miR-217 expression by FAK inhibitors indicates that miR-217 is involved in the process of EGF-induced EMT in colorectal cancer cells. This study is the first to report the presence of the binding site AUGCAGU for miR-217 in the 3′ UTR region of E-cadherin, which precisely matches the UACGUCA sequence in miR-217, providing a theoretical basis for miR-217 as a potential target for tumor inhibition.

The Caco-2 cell line was selected for this study. Caco-2 cells are a human-cloned colon adenocarcinoma cell, similar in structure and function to differentiated small intestinal epithelial cells, with microvilli and other structures, and containing enzyme systems related to the small intestinal brush border epithelium. It can be used for experiments that simulate intestinal transport in vivo. Under cell culture conditions, cells growing on porous permeable polycarbonate membranes can fuse and differentiate into intestinal epithelial cells, forming a continuous monolayer, which is counterdifferentiated from normal mature small intestinal epithelial cells during in vitro cultivation. The situation is different. Cell sub-microstructure studies have shown that Caco-2 cells are morphologically similar to human small intestinal epithelial cells, with the same cell polarity and tight junctions. Although Caco-2 cells are similar to human small intestinal epithelial cells since Caco-2 cells are derived from the human colon, and their transport characteristics, enzyme expression, and transmembrane resistance are relatively more reflective of colon cells than small intestinal cells. At the same time, we will add cell lines with different characteristics in future research.

In summary, EMT in colorectal cancer cells is a synergistic process involving multiple factors. EGF can induce EMT in these cells, and the kinase FAK is also involved. The specific mechanism of this process involves the binding of EGF to EGFR, causing FAK phosphorylation, upregulation of miR-217 expression, and downregulation of E-cadherin; this induces EMT and enhances the migrating ability of colorectal cancer cells. The results showed that upregulation of E-cadherin expression and downregulation of vimentin expression via EGF was inhibited by FAK kinase inhibitor, PF-228. However, it is unclear that these PF-228-regulated molecule expressions were through transcription or posttranslation modification mechanisms. So, the definite mechanism about these PF-228-regulated factors might be explored in our future work. Furthermore, it might need further exploration whether PF-228 exerts the inhibition of migration and invasion through other signaling pathways. This clarification of the mechanism underlying the metastasis of colorectal cancer provides a theoretical basis for the identification of suitable targets for the treatment of colorectal cancer. However, the definite molecular mechanism about EGF/FAK/EMT will be explored in future.

## Data Availability

The datasets used and/or analyzed during the present study are available from the corresponding author upon request.
